# Navigating the Hazards: A Case Study on the Complexities of Battery Ingestion in an Adult

**DOI:** 10.1002/ccr3.70184

**Published:** 2025-02-10

**Authors:** Ashujot K. Dang, Prithi Choday, Carlos Buitrago, George Saffouri

**Affiliations:** ^1^ Division of Internal Medicine University of California, Riverside Riverside California USA; ^2^ Department of Internal Medicine, Hemet Global Medical Center Hemet California USA; ^3^ Department of Gastroenterology University of California, Riverside Riverside California USA

**Keywords:** battery ingestion, colonoscopy retrieval, endoscopic management, foreign body ingestion, mental health comorbidities, recurrent foreign body ingestion

## Abstract

This case underscores the potential for prolonged colonic retention of ingested batteries (even over a month) necessitating endoscopic retrieval. Early detection, prompt intervention, and preventive measures are crucial to prevent life‐threatening complications in high‐risk patients. Additionally, outpatient scheduled follow‐ups and management of recurring ingestions may help improve patient outcomes.

## 1 Introduction

Foreign body (FB) ingestion is a frequent reason for hospital emergency department visits, especially among children [1]. In adults, patients with underlying psychiatric conditions, prisoners, the elderly, and alcohol‐intoxicated individuals account for an overwhelming majority of these cases [2, 3]. Recent literature indicates that while most foreign objects are able to pass through the gastrointestinal tract without harm, certain high‐risk cases necessitate endoscopic procedures to safely extract them [4].

The management of FB ingestion, particularly in adults, presents unique challenges and continues to remain a subject of debate. According to recent American Society for Gastrointestinal Endoscopy (ASGE) guidelines, high‐risk ingestions include objects longer than 5 cm or wider than 2.5 cm, sharp objects, batteries, and magnets [5]. The high‐risk adult population is more prone to ingesting these objects, including batteries. Controversy lies in the optimal management and timing of endoscopic retrieval among these high‐risk cases. Some literature supports conservative management, while other studies advocate for early endoscopic intervention to avoid complications, including obstruction and perforation [4].

We present a report detailing a case of an adult with recurrent battery ingestions over the past several years, necessitating repeated endoscopic retrieval of the battery and highlighting the importance of prompt recognition. The case emphasizes methods to improve outcomes in high‐risk individuals, including a decrease in inpatient readmissions and a reduction in health care costs.

## 2 Case History

A 44‐year‐old female with a past medical history of recurrent foreign body ingestion, bipolar disorder on lithium therapy, antisocial personality disorder, schizoaffective disorder, seizure disorder, and class III obesity was brought to the emergency department from an institutional facility for the ingestion of multiple AA batteries. The patient reported battery ingestion 1 month before admission and was seeking medical care as she believed she did not pass the batteries in her stool. She had 12 prior hospital admissions for the ingestion of AA and AAA batteries from handheld radio sets over the prior 7 years. Abdominal imaging performed on previous admissions showed batteries at different locations in the stomach and throughout the colon, most of which resulted in natural excretion (sometimes with laxative administration). The patient was not taking any medications prior to admission. Upon arrival at the hospital, the patient was hemodynamically stable and in no acute distress. The physical exam was benign except for mild generalized tenderness to palpation of the abdomen. Upon reviewing the patient's outpatient records, an abdominal X‐ray obtained 1 month prior to admission showed a foreign body in the shape of an AA battery located in the ascending colon.

## 3 Methods (Differential Diagnosis, Investigations, and Treatment)

**FIGURE 1:**
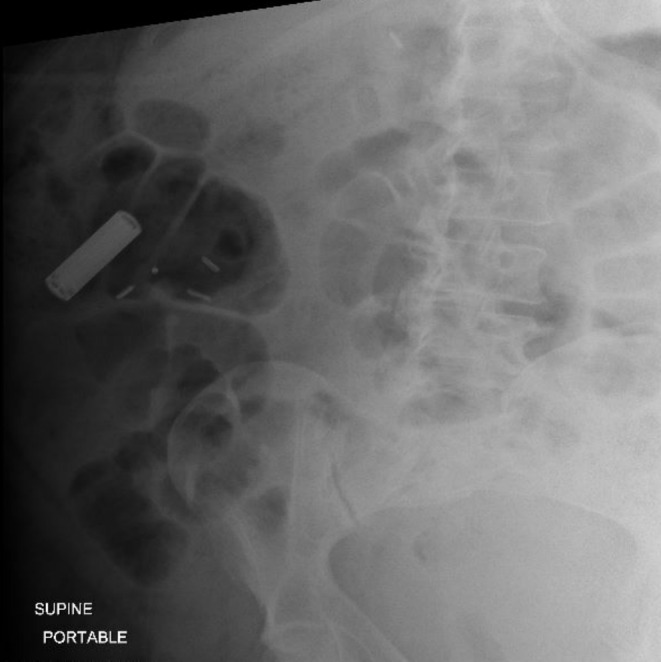
Abdominal X‐ray showing a foreign body seen on the right side of the abdomen.

**FIGURE 2:**
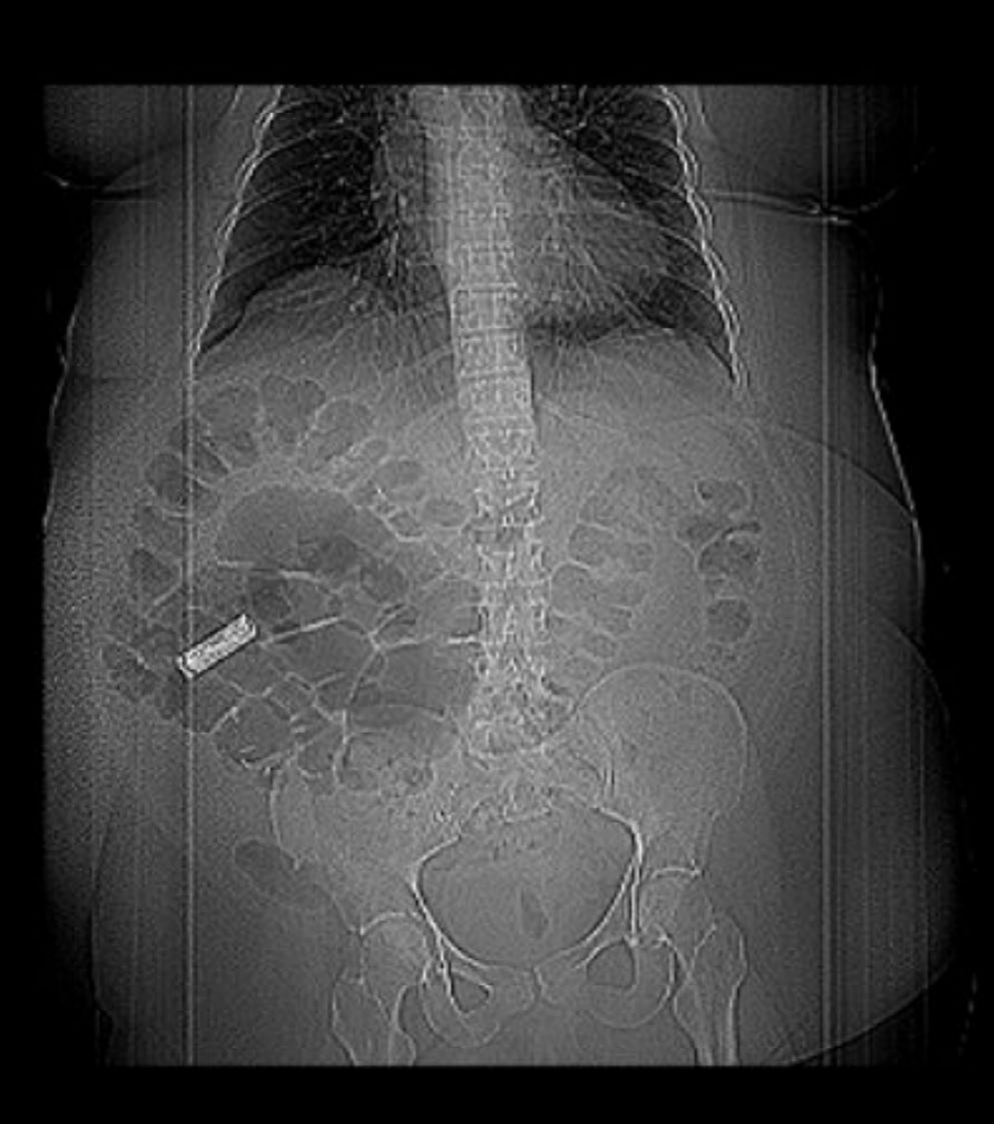
Computed tomography (CT) of the abdomen and pelvis confirmed a radiopaque foreign body measuring approximately 4 cm in length within the lumen of the ascending colon.

Laboratory studies revealed an unremarkable complete blood count and complete metabolic panel, and a low lithium level of 0.4 mEq/dL (0.6–1.2 mEq/L) [6]. The electrocardiogram showed normal sinus rhythm. Abdominal X‐ray on admission revealed a radiopaque foreign body overlying the right lower quadrant with the shape and consistency of an AA battery (Figure 1). Computed tomography (CT) of the abdomen and pelvis confirmed a radiopaque foreign body measuring approximately 4 cm in length within the lumen of the ascending colon (Figure 2). Gastroenterology was consulted and recommended serial X‐rays and inpatient colonoscopy after informed consent.

**FIGURE 3:**
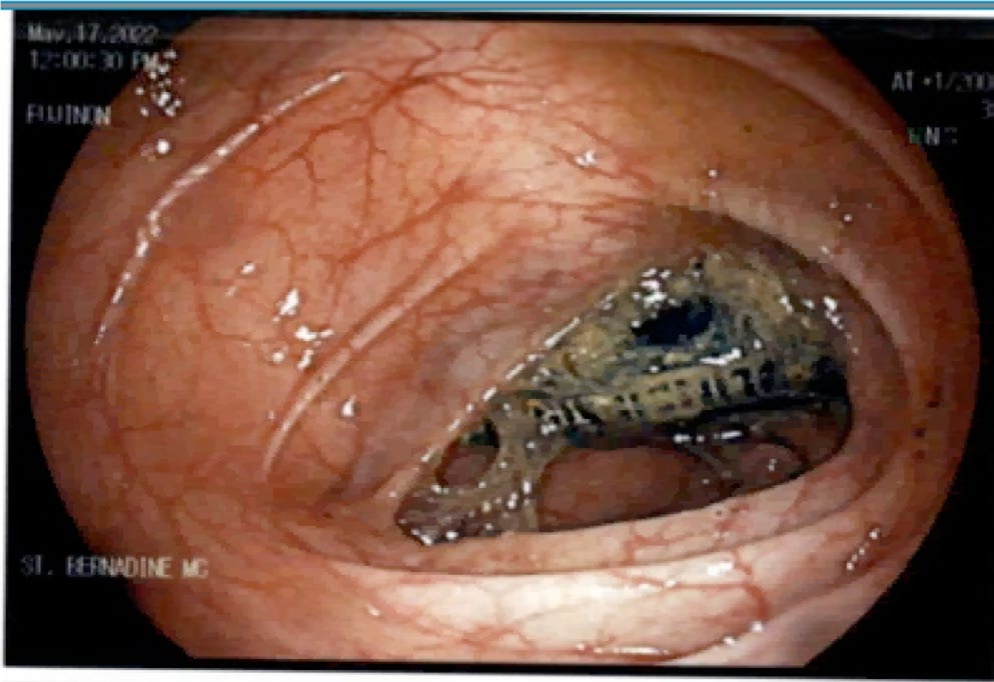
Battery lodged in the ascending colon with adherent food and stool.

**FIGURE 4:**
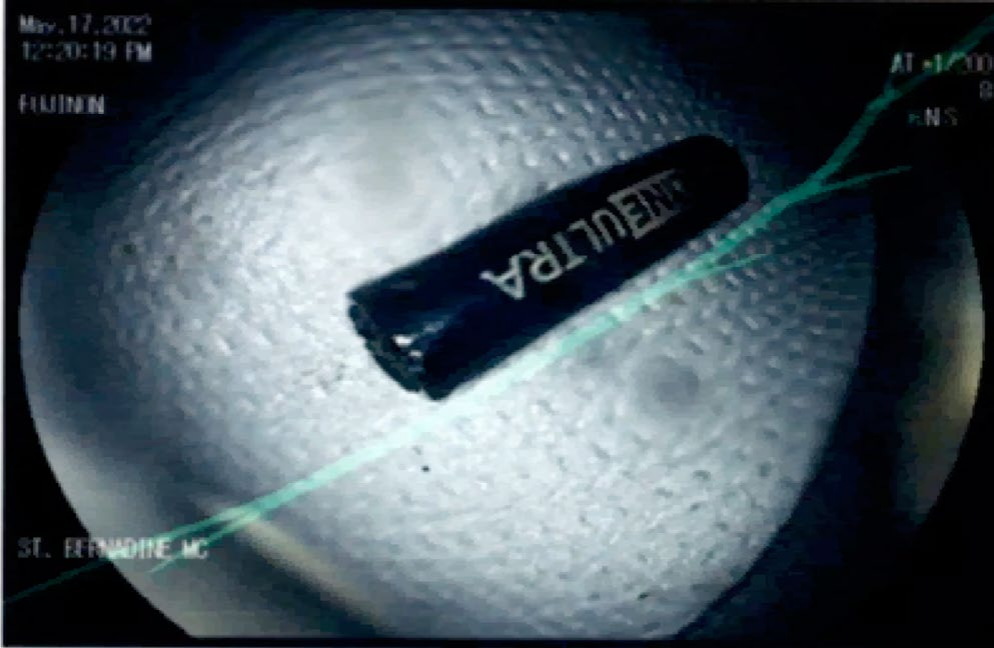
AA battery after colonoscopic retrieval with battery casing still intact.

Following adequate bowel prep with polyethylene glycol, colonoscopy was performed under monitored anesthesia care. A rectal examination was performed, which was normal. Given the difficult anatomy of the patient as noted on previous procedure reports, a pediatric colonoscope was used for improved flexibility and access. The colonoscope was inserted through the patient's anus and advanced to the ascending colon. The prep was good throughout the colon. An alkaline battery was visualized in the ascending colon encased by food particles and stool (Figure 3) This was dislodged using a biopsy forceps and a snare. The slipperiness of the battery made it challenging to grasp with a snare. A retrieval net was therefore employed to successfully extract the battery from the ascending colon. The battery casing was intact and there was no evidence of leakage of the internal contents (Figure 4). The colonoscope was then reinserted to evaluate the ascending colon. No ulcerations, lesions, or bleeding were found. The remainder of the endoscopic exam was normal.

## 4 Results (Outcome and Follow‐Up)

**FIGURE 5:**
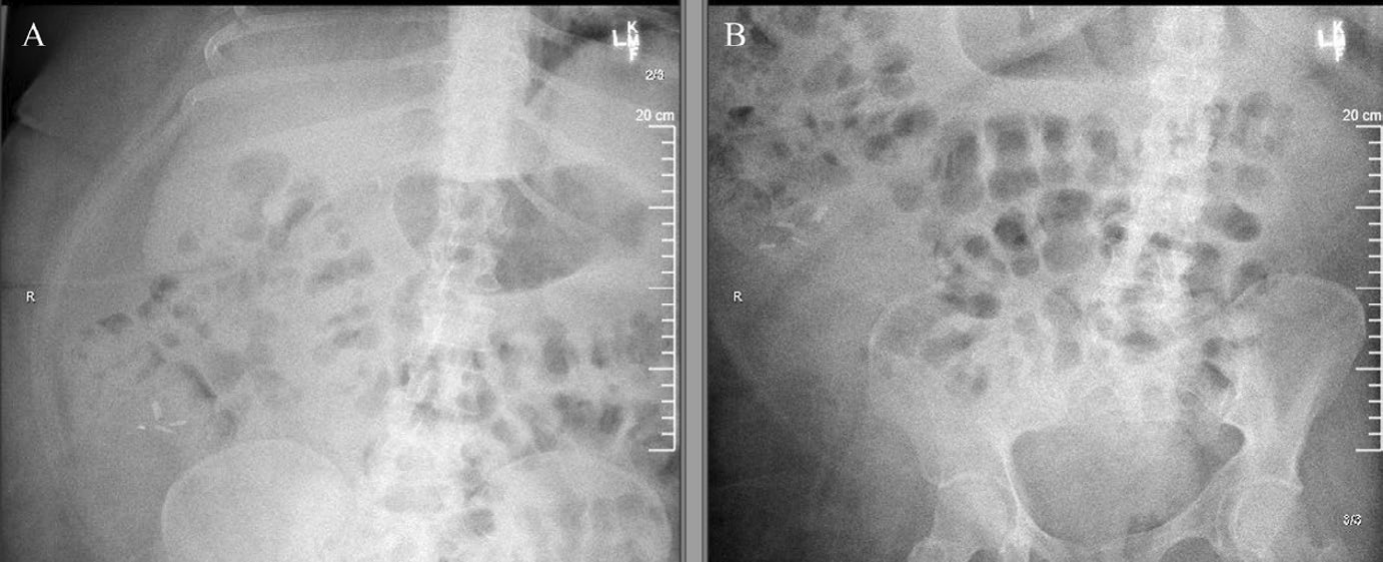
Abdominal radiographs obtained after retrieval. (A) The left image demonstrates post‐colonoscopic retrieval, confirming the successful removal of the foreign body. (B) The right image further highlights the successful retrieval, with evidence of normal bowel gas patterns and no signs of obstruction or perforation.

The patient tolerated the procedure well and was allowed to resume a regular diet later that day. Repeat X‐ray the following morning confirmed the removal of the foreign body (Figure 5). Education was provided on the potential hazards of further ingestions, particularly high‐risk objects. Additional recommendations were provided to the facility in an attempt to avoid further FB ingestions. The post‐procedural course was uneventful, symptoms improved, and the patient was discharged the following day.

## 5 Discussion

Foreign body ingestion is a common cause of emergency department visits worldwide, with the most common being button battery ingestion by children [7]. A large retrospective study using pooled data from the National Poison Data System, National Battery Ingestion Hotline, and a meta‐analysis of relevant medical literature found that 56,535 cases of battery ingestion were reported between 1985 and 2009 in the United States [8]. These data reveal a 6.7‐fold increase in the percentage of button battery ingestions with major or fatal outcomes from 1985 to 2009. Specifically, ingestion of 20‐ to 25‐mm diameter button batteries increased from 1% to 18%, and ingestion of lithium or cylindrical batteries increased from 1.3% to 24% during this period. Additionally, O'Sullivan et al. [9] studied the increasing incidence of deliberate foreign body ingestion amongst institutionalized psychiatric hospital patients and prison inmates, according to a retrospective study of 36 patients. Extra caution should be taken in these communities due to the higher incidence of biting the ends of batteries prior to ingestion in order to increase the toxic potential. The increasing incidence among the population as a whole and in these select communities should be of special concern among clinicians.

Though 80% of ingested foreign bodies pass spontaneously without intervention [5, 10], ingestion of cylindrical batteries is of particular concern due to the potentially harmful sequelae associated with it. Outcomes were shown to be significantly worse for large‐diameter lithium cells (> 20 mm) and in small children [8]. The large size of the cylindrical batteries can put a patient at high risk of lodgement, intestinal obstruction, perforation, and hemorrhage. Additionally, leakage of dangerous alkaline battery contents, including zinc, manganese, mercury, and lithium, can lead to peritonitis, tissue erosion, and even death. Several cases of mercury toxicity from alkaline battery ingestion have been reported [11, 12]. If toxicity is suspected, the National Poisons Information Service should be notified using their primary database, TOXBASE. Cylindrical batteries lodged within the lumen of the colon that have not been successfully excreted with laxatives require endoscopic or surgical intervention to retrieve them. Two recent studies have shown that in the setting of intentional ingestion, the rate of endoscopic intervention was much higher (63%–76%) compared with those with unintentional ingestion [5, 10]. Unfortunately, even after removal of the ingested battery, perforation, tracheoesophageal fistulas, fistulization into major vessels, massive hemorrhage, and even death have still been shown to occur, particularly with lithium button batteries, less so with alkaline batteries [8]. Alkaline battery ingestions are additionally at risk for caustic injuries like corrosive esophageal ulcerations from the alkaline battery contents [13] and electrothermal injury if the battery case is ingested intact [14]. American Society for Gastrointestinal Endoscopy (ASGE) Guidelines briefly address cylindrical battery ingestion, with emergency management in instances of esophageal obstruction or with a compromised battery casing. Per the ASGE, intact cylindrical batteries should be removed in the event that they remain in the stomach for more than 48 h [5]. Despite this recommendation, there are data demonstrating reassuring conservative medical management with batteries remaining in the stomach for more than 48 h [15]. The optimal strategy—be it medical or interventional—and the possibility of catastrophic events warrant further investigative studies.

This case report highlights the complexities and challenges associated with the management of foreign body ingestion, particularly in cases involving cylindrical battery ingestion. The patient in our report had an extensive history of AA and AAA battery ingestions over the prior 7 years. She also had a long history of psychiatric illness, including bipolar disorder, schizoaffective disorder, and antisocial personality disorder, and was institutionalized with easy access to handheld radio batteries, putting her at a higher risk for intentional battery ingestion [9]. Of note, despite the battery having been lodged in her ascending colon for nearly 1 month, the patient did not present with any distress or gastrointestinal symptoms. The potential deleterious effects of cylindrical battery lodgement, including obstruction, perforation, and leakage of contents, necessitated urgent and careful management with endoscopic removal. It is imperative for clinicians to be aware of the potential harmful effects of battery ingestion and pursue appropriate management to prevent deleterious outcomes for their patients.

Methods of prevention of future foreign body ingestions for this patient may include appropriate psychiatric counseling and management, caution with access to battery‐operated items, and education of the facility staff. Adult patients with a history of complicated and repetitive foreign body ingestions may initially be given a trial for spontaneous passage with the aid of laxatives if needed, but if unable to pass the high‐risk objects, they should undergo endoscopic retrieval (Table 1) We propose a checklist for outpatient management of battery ingestions (Table 2). Although catastrophic complications from cylindrical alkaline batteries are rare, given the possibility of obstruction, perforation, or peritonitis, we propose patients undergo outpatient endoscopic retrieval if feasible. This would likely require coordination and an established care model with high‐risk settings (e.g., prisons, psychiatric facilities) and outpatient GI labs. It seems prudent to consider endoscopic management within 1 week of ingestion, especially if the patient is symptomatic or there is evidence of the battery not moving through the tract, though this can certainly be assessed on a case‐to‐case basis. The outpatient setting can help reduce hospital (re)admissions, improve morbidity and mortality, and decrease overall healthcare costs. Given the complexities and caveats surrounding the management of cylindrical battery ingestions, there are few data or studies to guide management. This subject demands more discussion and review to formulate updated, specific guidelines for optimal and timely management.

**TABLE 1:** High‐risk groups and the foreign objects they are likely to ingest.

High risk individuals	High risk objects
Psychiatric disorders	Sharp objects
Prisoners	Batteries
Elderly	Objects longer than 5 cm or wider than 2.5 cm
Alcohol intoxicated	Magnets

**TABLE 2:** Checklist in battery ingestion to guide community clinicians.

Checklist to guide community clinicians
Monitoring clinical symptoms
Weekly X‐rays
Serial abdominal exams
Additional imaging as needed if symptoms/signs worsen
Early intervention if high risk

## 6 Conclusions

This case emphasizes the critical need for heightened awareness among healthcare providers regarding the risks and management of cylindrical alkaline battery ingestion among high‐risk adults, which is not as common as button battery ingestions. We propose methods to systematically evaluate and manage patients in the hopes of improving outcomes and reducing strain on the healthcare system. Further studies are essential to elucidate underlying disparities contributing to these incidents and develop effective preventive strategies. A better understanding of these cases can help future clinicians identify people at risk, implement targeted interventions, and ultimately improve the incidence and outcomes of battery ingestions.

## Author Contributions

**Ashujot K. Dang:** writing – original draft, review and editing. **Prithi Choday:** writing – review and editing. **Carlos Buitrago:** writing – review and editing. **George Saffouri:** writing – review and editing.

## Disclosure

The authors have nothing to report.

## Ethics Statement

The authors have nothing to report.

## Consent

Informed written consent was obtained from the patient to publish this report in accordance with the journal's patient consent policy.

## Conflicts of Interest

The authors declare no conflicts of interest.

## Data Availability

The authors have nothing to report.
